# Cellular Functions Regulated by Phosphorylation of EGFR on Tyr845

**DOI:** 10.3390/ijms140610761

**Published:** 2013-05-23

**Authors:** Ken-ichi Sato

**Affiliations:** Laboratory of Cell Signaling and Development, Department of Molecular Biosciences, Faculty of Life Sciences, Kyoto Sangyo University, Kamigamo-Motoyama, Kita-ku, Kyoto 603-8555, Japan; E-Mail: kksato@cc.kyoto-su.ac.jp; Tel.: +81-75-705-2916.

**Keywords:** apoptosis, cancer, cell proliferation, EGF, mitochondria, phosphorylation, signal transduction, Src

## Abstract

The Src gene product (Src) and the epidermal growth factor receptor (EGFR) are prototypes of oncogene products and function primarily as a cytoplasmic non-receptor tyrosine kinase and a transmembrane receptor tyrosine kinase, respectively. The identification of Src and EGFR, and the subsequent extensive investigations of these proteins have long provided cutting edge research in cancer and other molecular and cellular biological studies. In 1995, we reported that the human epidermoid carcinoma cells, A431, contain a small fraction of Src and EGFR in which these two kinase were in physical association with each other, and that Src phosphorylates EGFR on tyrosine 845 (Y845) in the Src-EGFR complex. Y845 of EGFR is located in the activation segment of the kinase domain, where many protein kinases contain kinase-activating autophosphorylation sites (e.g., cAMP-dependent protein kinase, Src family kinases, transmembrane receptor type tyrosine kinases) or trans-phosphorylation sites (e.g., cyclin-dependent protein kinase, mitogen-activated protein kinase, Akt protein kinase). A number of studies have demonstrated that Y845 phosphorylation serves an important role in cancer as well as normal cells. Here we compile the experimental facts involving Src phosphorylation of EGFR on Y845, by which cell proliferation, cell cycle control, mitochondrial regulation of cell metabolism, gamete activation and other cellular functions are regulated. We also discuss the physiological relevance, as well as structural insights of the Y845 phosphorylation.

## 1. Introduction

### 1.1. Discovery of Src and EGFR, and Their Crosstalk

Protein-tyrosine phosphorylation was initially discovered as the catalytic property of the viral oncogene product v-Src [1,2]. Its cellular homologue, namely, the protein product of the c-Src gene (Src), also possesses the activity of a protein-tyrosine kinase (PTK). While the activity of v-Src is deregulated (*i.e.*, constitutively activated) and thus contributes to malignant cell transformation, the activity of the normal cellular Src is usually negatively regulated and its signal-dependent transient activation contributes to physiological cell responses (e.g., proliferation, differentiation, cell motility) [3–5]. Src is also recognized as the first example of a cytoplasmic as well as membrane-associated PTK, whose structural organization is shared by several other proteins, collectively termed the Src family PTKs [6]. Src and other Src family PTKs are expressed ubiquitously (e.g., Src, Fyn and Yes) or in certain specific tissues (e.g., Lck in lymphoid cells), exerting both common and specific functions.

**Figure 1:**
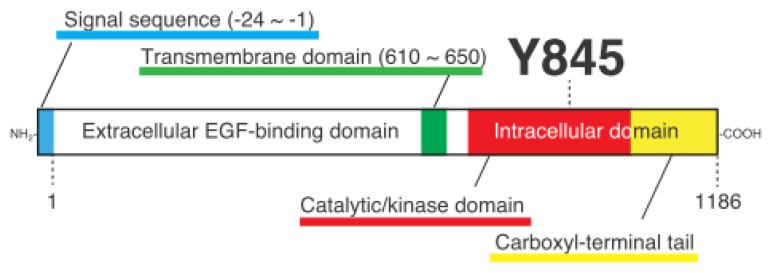
Schematic structure of EGFR. The translational protein product of the human EGFR consists of a single polypeptide of 1210 amino acids. Upon protein maturation that involves extensive glycosylation of the amino-terminal EGF-binding extracellular domain, the amino-terminal 24 amino acids are removed as a signal sequence (as indicated by a blue area), by which the mature protein becomes a polypeptide of 1186 amino acids. In addition to the extracellular domain, EGFR contains a single transmembrane domain at almost the center of the protein (green) and a carboxyl-terminal sequence that contains the catalytic/kinase domain (red) and a non-catalytic tail sequence (yellow), between the two where the Src phosphorylation site Y845 and several tyrosine residues to be autophosphorylated are located.

The epidermal growth factor receptor (EGFR), on the other hand, was identified as the first example of a transmembrane receptor/PTK whose activation depends upon the binding of extracellular ligands (EGF and other EGF-like ligands, such as transforming growth factor-α, heregulin, heparin-binding EGF-like growth factor) to and dimerization of the receptor molecules [7] (Figure 1). EGFR also constitutes a family of proteins, named the HER family in humans, which includes EGFR/erbB1/HER1, erbB2/HER2/Neu, erbB3/HER3 and erbB4/HER4, which can form homologous (e.g., EGFR-EGFR) as well as heterologous dimers (e.g., EGFR-HER2) exerting specific functions as highly specific ligand-receptor interaction [8]. It is also known that v-erbB, a product of avian erythroblastosis virus AEV-H, is the oncogenic version of EGFR/erbB1, in which the *N*-terminal ligand-binding sequence is almost entirely truncated and thus the PTK activity of the protein is deregulated [7,9]. The function of EGF and EGFR was initially documented in epidermal cellular physiology; however, later studies showed that they are ubiquitously expressed in a variety of cells and tissues, and contribute to a variety of cellular functions [10].

After the discovery of Src and EGFR, much work has focused on understanding how PTKs and their substrates act as a trigger of cellular functions, including malignant cell transformation. These studies include analysis of physical and/or functional interactions, that is, crosstalk, between Src and transmembrane receptor/PTKs such as platelet-derived growth factor receptor [11], Trk/nerve growth factor receptor [12], colony-stimulating factor receptor [13], erbB2/HER2/Neu [14], and EGFR [15,16]. In particular, the interaction of Src and EGFR has raised much interest because both are ubiquitously expressed and their co-overexpression and/or mutations have often been found in cancer cells of humans and model animals.

### 1.2. Auto- and Trans-Phosphorylation of EGFR

Soon after the molecular identification and cDNA cloning of EGFR performed in the mid-1980s, several tyrosine residues that reside in the carboxyl-terminal tail were identified as the autophosphorylation sites in activated EGFR (for reviews, see [17]) (Figure 1). In addition, several research groups demonstrated the importance of these phosphotyrosine residues in EGF-induced signal transduction, namely, as docking sites for phosphotyrosine-binding adaptor proteins such as Grb2 and Shc, in the early 1990s [18]. We demonstrated for the first time the occurrence of the EGFR-Y845 phosphorylation in 1995 [19], and the Parsons group demonstrated for the first time the physiological as well as pathological importance of the Y845 phosphorylation in 1999 [20,21].

Ligand-activated EGFR undergoes autophosphorylation on multiple tyrosine residues, most of which are located in the *C*-terminal non-catalytic sequence; they include Y992, Y1068, Y1086, Y1148, and Y1173 [7,22–26]. Later studies showed that tyrosine residues in EGFR could also be phosphorylated by other kinases including Src (Y845, for detail see below; Y891 and Y920 [27]; Y1045 [28]; Y1101 [20]; Y1173 [29]). The phosphorylation of serine and threonine residues in EGFR was also found: it included T654, T669, S967, S971, S1002, and S1046/S1047 [15,16,30–32]. Several lines of evidence indicate that the autophosphorylated tyrosine residues serve as docking sites for a variety of signaling molecules that contain a phosphotyrosine-binding sequence (*i.e.*, Src homology 2 or phosphotyrosine-binding domains). On the other hand, serine/threonine phosphorylation acts as a regulatory mechanism for the dimerization, catalytic activity, and/or turnover of the protein [8].

In many protein kinases so far identified and biochemically characterized, a tyrosine and/or serine/threonine residue(s) in a segment, which resides between kinase subdomains VII and VIII (often called the “activation segment”), serves as an autophosphorylation and/or trans-phosphorylation site(s) [33,34], a well-known example of which is Y416/Y418 in the chicken/human Src protein. However, a homologous tyrosine residue that is present in the activation segment of EGFR, that is, Y845 (or Y869, if the *N*-terminal signal sequence of 24 amino acids is included) (Figure 1), was not identified as the phosphorylation site in the early years of EGFR study. Against this background, initial characterization of Y845 phosphorylation was made by *in vitro* kinase assays using a synthetic peptide straddling Y845 (E-E-K-E-Y845-H-A-E) and mutated forms of it [35]. Subsequently, Gotoh *et al*. [36] made an initial evaluation of the role of Y845 in EGFR function, by which the Y845F mutant of EGFR was shown to be able to transform NIH3T3 cells and undergo autophosphorylation. This result suggests that, at least in the NIH3T3 cell system, Y845 is dispensable for cellular functions regulated by EGFR. However, a growing body of knowledge indicates that Y845, through its trans-phosphorylation rather than autophosphorylation, plays a pivotal role in several aspects of cellular functions involving EGFR, a main mediator of which is the tyrosine kinase Src that catalyzes the phosphorylation of Y845.

### 1.3. Discovery of Y845 Phosphorylation of EGFR by Src

Intracellular communication between EGFR and Src, which contributes to cancer malignancy, was first demonstrated in the late 1980s. Parsons and others showed that EGF-dependent protein-tyrosine phosphorylation and mitogenic cell responses are augmented in cells overexpressing Src [16,37,38]. At this time, however, phosphorylation of Y845 by Src was unknown. Later, Wasilenko *et al*. [39] made the first demonstration that Src is capable of phosphorylating EGFR on unknown tyrosine residue(s). In this work, trypsin-digested phosphopeptides were analyzed in cells expressing both EGFR and Src, and thus the identification of phosphorylated Y845 was not made. The first evidence showing that Src directly phosphorylates EGFR on Y845 was provided by our work [19], where *in vitro* phosphorylated EGFR in the presence of Src was analyzed by tryptic digestion and two-dimensional electrophoresis, by which the identities of EGFR-derived phosphopeptides and a synthetic peptide containing phosphorylated Y845 were evaluated. In addition, we showed that Y845 phosphorylation also occurs in A431 epidermoid carcinoma cells, where a fraction of EGFR and Src constitutes a physical complex via the activation segment of Src [19,40] that we called the inter-DFG-APE region [33,41]. At almost the same time, Parsons and others demonstrated that phosphopeptide maps, analyzed after preparation from C3H10T1/2 murine fibroblast-derived 5H cells overexpressing Src, contain an Src-dependent phosphopeptide that is suggested to contain Y845 [42]. Later, the same group showed that EGFR is phosphorylated on Y845 and Y1101 in 5H cells [20]. Stover *et al*. also showed that EGFR is phosphorylated on two novel tyrosine residues in DLD-1 colorectal cancer cells and MCF-7 breast cancer cells: in this case, Y891 and Y920, but not Y845, were identified [27]. These studies suggest that EGFR and Src communicate with each other by Src-dependent phosphorylation of novel tyrosine residues (for review, see [4,43–46]).

## 2. Y845 Phosphorylation and Cancer Cell Functions

### 2.1. Cooperation of EGFR and Src in Cancer Cells Involving Y845 Phosphorylation

As described above, elevated levels of Src and EGFR are often found in a variety of cancer cells [42]. Search of the physiological roles played by these two overexpressed PTKs has demonstrated a breakthrough finding that they exert synergism in promoting as well as maintaining cancerous cell growth, the main phenomenon implicated in which is Y845 phosphorylation of EGFR by Src [21]. It was shown that the activity of Src is required for C3H10T1/2 fibroblast cells overexpressing EGFR to undergo cell transformation. As opposed to the case using an NIH3T3 cell system ([36], see above), expression of Y845F mutant EGFR in this cell line leads to a failure of DNA synthesis in response to not only EGF but also serum and lysophosphatidic acid, indicating that the Y845F mutant has a dominant-negative effect on the cellular function of EGFR [21]. In this case, however, the Y845F mutant is still capable of interacting with Src. In addition, it was demonstrated that neither stimulation of the kinase activity of EGFR nor that of extracellular signal-regulated kinase (ERK)/mitogen-activated protein kinase (MAPK) was affected by the expression of the Y845F mutant, suggesting that an unknown, non-canonical signaling pathway contributes to the alteration of EGFR molecular function, DNA synthesis, and malignant cell proliferation under the control of Y845 phosphorylation [20,21].

Subsequent studies using breast cancer and other types of human cancer cells demonstrated that the signal transducer and activator of transcription protein (STAT) is a possible mediator of the Y845 phosphorylation-dependent synergism of EGFR and Src [47–49]. In breast cancer cells, STAT5b was identified as a prominently tyrosine-phosphorylated protein and that expression of the Y845F mutant of EGFR has an inhibitory effect on this event [47]. The importance of STAT5b is further demonstrated by the fact that the tyrosine phosphorylation-defective mutant of STAT5b is inhibitory to DNA synthesis and proliferation in cells expressing EGFR and Src [47,50,51]. On the other hand, STAT1 and 3, but not STAT5b, have been identified as tyrosine-phosphorylated proteins in EGF-stimulated A431 cells [49], in which the physical interaction of EGFR and Src involving Y845 phosphorylation has been demonstrated [19,40,48,52]. In this system, it has been shown that high concentrations of EGF or the expression of the Shc adaptor protein p52/p66, a direct regulator of the Src activity [48,53], promote cell cycle arrest and apoptosis, which is accompanied by the induction of p21^waf1^[54,55]. The application of a Src-specific inhibitor PP2 or an anti-Y845 phospho-specific antibody into the cells results in decreases in the EGF-dependent phosphorylation of STAT1/3 and in the extent of p21^waf1^ induction [49], suggesting that Src-dependent Y845 phosphorylation serves as a pro-apoptotic signal in this system.

The group of Parsons also showed that cytochrome c oxidase subunit II (CoxII) is an alternative binding partner of the phosphorylated Y845 of EGFR [50]. CoxII is a mitochondrion-associated protein that acts as a component of the oxidative phosphorylation pathway. Mitochondria are well known as the center for energy metabolism as well as survival machinery. The importance of Y845 phosphorylation and CoxII engagement of EGFR in mitochondria is suggested by the fact that wild-type EGFR, but not Y845 mutant of EGFR, translocates to mitochondria where it physically associates with CoxII, and that such interactions contribute to an increase in survival of MDA-MB-231 breast cancer cells under serum-deprived or adriamycin-treated “pro-apoptotic” conditions [56,57]. A growing body of knowledge indicates that Src can also be localized to mitochondria [58–64] (for review, see [65]) and that Src may be responsible for maintaining cell survival through phosphorylation of mitochondrial proteins [66], suggesting that EGFR and Src communicate not only in the plasma membrane but also in cytoplasmic organelles. In addition, a mutant form of EGFR expressed in glioma cells, named de2-7EGFR/EGFRvIII, was shown to translocate to mitochondria in an Src-dependent manner and to be phosphorylated on Y845 [67]. More recently, Miyake and Parsons [68] have shown that choline kinase α, an enzyme that converts choline to phosphocholine in the phosphatidylcholine synthesis and whose overexpression correlates with poor prognosis, high grade and increased aggressiveness in some types of human cancer, interacts directly with EGFR, is tyrosine-phosphorylated in an Src-dependent manner, and contributes to breast cancer cell proliferation. However, the importance of Y845 phosphorylation in this process is not known.

### 2.2. Y845 Phosphorylation in Several Types of Cancer Cells

In hepatocellular carcinoma, where transmembrane 4 L six family member 5 (TM4SF5), a tetraspanin-type protein, is implicated in their migration and invasion, a high incidence of Y845 phosphorylation of EGFR was demonstrated [69]. Later studies by Jung *et al*. [70] showed that TM4SF5 is responsible for the recruitment of negatively regulated Src to the vicinity of EGFR, by which Src becomes activated and phosphorylates Y845 of EGFR, and cells acquire the ability to form invasive protrusions that involve actin and cortactin interactions. A similar mechanism of activation of the Src-EGFR pathway was also reported in breast epithelial cells, where expression of translationally controlled tumor protein (TCTP) results in a release of inactive Src from its interaction with Na^+^, K^+^-ATPase α subunit, so that Src becomes activated and induces cell transformation involving Y845 phosphorylation [71,72].

EGFR activation is often accompanied by the redistribution of EGFR from the plasma membrane to intracellular vesicles [73]. This event involves endocytosis of the receptor via clathrin-coated vesicles and endosome formation. Such endocytic internalization of EGFR can be augmented by overexpression [74] and temperature shift-dependent activation of Src (ts/v-Src) [75] in C3H10T1/2 murine fibroblasts and MDCK/tsLA31 cells, respectively. In the latter case, the occurrence of Y845 and Y1173 phosphorylation, in conjunction with the endosomal distribution of EGFR, is demonstrated [75].

There is a specific type of EGFR mutation, called de2-7EGFR or EGFRvIII, whose expression is often found in glioma or glioblastoma multiforme (GBM). In this case, Src-dependent phosphorylation of Y845 seems to be important for the survival and proliferation of glioma cells through its involvement in the stimulation of mitochondrial oxidative metabolism under low-glucose conditions [67]. Actually, mitochondrial localization of this EGFR mutant was shown to depend upon Y845 phosphorylation. In another study using U373MG cells, another type of GBM cells, urokinase-type plasminogen activator receptor (uPAR) was shown to interact with EGFRvIII and to be important for Y845 phosphorylation and sustained ERK activation in this cell system [76]. Huang *et al*. [77] showed that, by mutating Y845 or two other tyrosine phosphorylation sites (Y1148 and Y1173), these specific tyrosine residues in EGFRvIII are required for suppressing the ERK activity, which acts against the cell proliferation of EGFRvIII-expressing U87MG glioblastoma cells.

Src also participates in cancer cell functions involving the other family member of the EGFR and/or its phosphorylation on a tyrosine residue that is analogous to the EGFR Y845. In murine fibroblasts, C3H10T1/2, and human breast cancer cells, MDA-MB-361, where high levels of expression of erbB2 (naturally overexpressed) and erbB3 (ectopically or naturally overexpressed) are evident, mutual interaction between erbB2 and erbB3 takes place. Such erbB2-erbB3 interaction was shown to be responsible for the erbB3-dependent (*i.e.*, the erbB3 ligand heregulin-dependent) anchorage-independent growth and cell motility in a Src-dependent manner [78]. In MBA-MB-361 cells, the heregulin-dependent cell functions involve the phosphorylation of erbB2 on Y877, a tyrosine residue analogous to EGFR Y845 [78]. Thus, Src-dependent phosphorylation of a tyrosine residue that resides in the activation segment is a phenomenon that occurs commonly for at least two EGFR family proteins, EGFR and erbB2. The incidence of EGFR-erbB2 interaction is also implicated as a prognostic parameter for high malignancy and drug resistance of breast cancer cells, and it is also associated with the occurrence of Y845 phosphorylation [79].

### 2.3. Y845 Phosphorylation as a Diagnostic Marker for Cancer Treatment

In the search for the molecular basis of drug resistance in patients with non-small-cell lung cancers (NSCLCs), Y845 and some other phosphorylation sites such as Y1045 and Y1068 have been identified as prominently phosphorylated sites in many gefitinib-resistant EGFR mutants [80,81]. Chung *et al*. [82] showed that Src-dependent Y845 phosphorylation in some mutated EGFR is critically important for the malignancy of NSCLCs. On the other hand, evaluation of the effect of gefitinib using human squamous cell carcinoma cells, UMSCC-1, demonstrated that this drug, in combination with gemcitabine (2′,2′-difluorodeoxycytidine), suppresses the phosphorylation of EGFR Y845 and arrests the cell cycle at the S or G1 phase, which leads to the increased incidence of apoptosis in the cells [83]. The occurrence of the EGFR mutation and its correlation with the appearance and/or the subcellular localization of Y845 phosphorylation have been examined in NSCLCs, adenocarcinomas [84], and breast cancer [85]. Sonnweber *et al*. [86] have shown that, in stage I NSCLCs, Y845 phosphorylation of EGFR, rather than the incidence of EGFRvIII mutation, has independent and highly predictive value for prognosis. In some cases of NSCLC expressing mutant EGFR, gefitinib does not inhibit cell proliferation, but PP2 does, collectively suggesting that gefitinib resistance of the mutant EGFR is due to the Src-dependent modulation of EGFR activity. In NSCLCs, the functional association of Y845 phosphorylation with the extent of autophosphorylation of co-expressing EGFR wild type and mutants (e.g., Y1068) and the sensitivity of the cells to Src-specific inhibitor PP2, have been demonstrated [87].

In breast cancers that are not responsive to EGFR inhibitor therapy, not only Src but also Met, the hepatocyte growth factor (HGF) receptor that is itself a tyrosine kinase, is implicated in cancer cell malignancy [88]. For example, in SUM229 breast cancer cells, Src activation and Y845 phosphorylation of EGFR depend upon the activity of Met, as judged by the effects of the Met-specific activator (HGF) and inhibitors (SU11274). Crosstalk of Met with the Src/EGFR signaling pathway has also been demonstrated in bladder carcinoma cells, in which serum-independent growth of the cells requires the activation of EGFR and Src, which leads to the phosphorylation of Met on Y1003 [89]. In this case, the phosphorylated Y1003 of Met, rather than the kinase activity of Met, seems to be responsible for the anti-apoptosis and proliferation under serum-starved culture conditions [89,90]. Whether or not Y845 phosphorylation takes place under serum-deprived conditions is unknown. In glioma cells, it has been shown that HGF stimulates the transcription of EGFR ligands such as transforming growth factor α (TGF α) and heparin-binding EGF-like growth factor (HB-EGF), whose inhibitions result in the failure of EGFR activation involving Y845 phosphorylation [91]. Thus, crosstalk between EGFR and Met is, at least in some cases, transcription-dependent.

Tamoxifen-resistant variants of MCF7 and T47D breast cancer cells are reported to exert Y845 signaling via the activation of insulin-like growth factor-I receptor (IGF-IR) in response to IGF-II [92]. The anti-invasive effect of oxidized streptolysin O on MDA-MB231 breast cancer cells is characterized by its ability to activate the autophosphorylation of EGFR selectively on tyrosine residues besides Y845, indicating that, in this case, Y845 may not be involved in the anti-cancer malignancy [93]. On the other hand, Y845 phosphorylation has been identified as a diagnostic marker for the inhibitory effect of ipriflavone on osteolytic bone metastasis of the same cells in a nude mouse model [94]. The prognostic value of Y845 phosphorylation has also been examined in oral squamous cell carcinoma; tumors bearing this phosphorylation correlated with a worse prognosis and were poor responders to chemotherapy [95]. In another case, where the effect of cetuximab, an anti-EGFR drug monoclonal antibody, on the growth of androgen-independent prostate cancer cells was evaluated, the drug-induced reduction of the Y845 phosphorylation did not necessarily correlate with the reduction in the growth potential of the cells [96], demonstrating that, under certain conditions, the Y845 phosphorylation of EGFR is dispensable for the EGFR-dependent cancer cell malignancy.

In colorectal cancer cells, sensitivity to an anti-EGFR drug (*i.e.*, gefitinib) can be improved by the pharmacological inhibition of insulin receptor isoform-A [97]. In this case, Y845, Y1068, and Y1173 are employed as diagnostic markers for the active state of EGFR. The anti-tumor effect of gefitinib and anastrozole has been investigated in estrogen-receptor-positive breast cancer cells, where the extent of Y845 phosphorylation is used as one of the outcomes of the drug treatments [98]. The anti-cancer pro-apoptotic function of an indole compound, 3,3′-diindolylmethane, toward ovarian cancer cells also involves the suppression of the Y845 phosphorylation of EGFR and the subsequent ERK/MAPK signaling pathway [99]. Specific inhibition of Y845 phosphorylation by the Src-specific inhibitor PP2 was also shown in cervical carcinoma cells HeLa and SiHa [100], and in ionizing irradiation-treated MDA-MB-468 breast cancer cells [101]. X-radiation-induced DNA synthesis in rat hepatocytes was additionally shown to involve Y845 phosphorylation [102].

Lu *et al*. [103] have reported the mechanism of resistance to the anti-EGFR monoclonal antibody cetuximab in colorectal cancer that had been made insensitive to this drug by exposure to subeffective doses of cetuximab over an extended period of time. In this system, the inhibition of Src activity and Y845 phosphorylation of EGFR reverses the drug resistance. An alternative explanation for the cetuximab resistance in breast cancer cells was reported by Li *et al*. [104], who showed that breast tumor kinase (Btk)/protein-tyrosine kinase 6 (PTK6), a non-receptor, as well as a non-Src family PTK, which was originally identified in human melanocytes, is highly expressed in most human breast cancers and that Btk/PTK6 seems to be responsible for the Y845 phosphorylation, the retention of which confers the resistance of cancer cells to an anti-EGFR drug, cetuximab. This was the first report describing that a non-Src family PTK can mediate Y845 phosphorylation.

Morgan *et al*. [105] showed that the combined use of the EGFR inhibitor cetuximab or erlotinib with gemcitabine increases the efficacy of radiation of damaging pancreatic cancers. In this case, phosphorylation of Y1173, but not Y845, was shown to be effectively decreased, indicating that Y1173, rather than Y845, is a target of these EGFR kinase inhibitors. In ovarian cancer cells that exhibit resistance to cisplatin therapy, NCX-4016, a nitro-derivative of aspirin, has been reported to inhibit effectively the EGFR signaling involving Y845 and Y992 phosphorylation of the receptor, and the activation of Akt and STAT3 [106]. Similar efficacy to EGFR signaling was also shown with the use of a cyclooxygenase-2 inhibitor, celecoxib, and a cyclic GMP phosphodiesterase inhibitor, exisulind, in the Wistar-Unilever rat prostate cancer model [107].

There are some alternative approaches to evaluate the EGFR Y845-dependent cancer cell conditions. Hudelist *et al*. [108] have shown that immunocytochemical detection of the phosphorylation of Y845 and other tyrosine residues in EGFR can be useful for predicting the clinical outcome of breast cancer patients undergoing anti-HER2/Neu antibody treatment. Proteomic tools such as two-dimensional gel electrophoresis and/or liquid chromatography-based protein separation and mass spectrometric determination of the protein structures are broadly used for “unbiased” identification of the molecules that participate in a complex array of the EGF-dependent signaling pathway. Phosphotyrosine-based proteome analysis has been employed in squamous carcinoma cells overexpressing EGFR (HN5) to elucidate the interactive networks involved in EGFR Y845 phosphorylation and activation, leading to the identification of its cross-talk relationship with not only well-characterized proteins, but also poorly described proteins such as desmoplakin3, a progesterone target of adhesion-related proteins [109].

## 3. Y845 Phosphorylation in a Variety of Cellular Functions

### 3.1. Y845 Phosphorylation in Transactivated EGFR

In HER2-overexpressing breast cancer cells, SK-BR-3, endothelin-induced transactivation of EGFR via phosphorylation of Y845 has been demonstrated, and dual targeting of the endothelin and EGFR systems, using atrasentan and trastuzumab, respectively, was shown to be effective in reducing cell proliferation and invasion [110]. In SK-BR-3 cells, it was also shown that macrophage inhibitory cytokine-1, a member of the TGF superfamily, transactivates EGFR and induces Y845 phosphorylation via Src activation [111]. Transactivation of EGFR by β2-adrenergic receptor signaling requires the phosphorylation of Y845 by Src to exert the full range of EGFR activation that is comparable to EGF-induced EGFR activation. In this particular case of transactivation, the priming of the EGFR activity via either the ligand stimulation or the overexpression of the receptor protein is also necessary [112]. Isoproterenol-induced transactivation of EGFR in astrocytes was also shown to involve Src-dependent Y845 phosphorylation; in this case, nanomolar concentrations, but not micromolar concentrations, of isoproterenol activation of β2-adrenergic receptor signaling are the trigger for this functional interaction, the astrocytic consequences of which are morphological differentiation and an increase in glial fibrillary acidic protein [113]. Transactivation of EGFR by α2-adrenergic receptor signaling in astrocytes in mature brain also involves Y845 phosphorylation [114].

In glioblastoma cell lines U-1242 MG and U-87 MG, phorbol 12-myristate 13-acetate (PMA), an activator of protein kinase C (PKC), has been shown to activate EGFR, as judged by the phosphorylation of multiple tyrosine residues (including Y845) [115]. In this transactivation system for EGFR, PKCδ-dependent activation of Src, which seems to require PKCδ phosphorylation of Src on serine 12 and/or serine 48, takes place. As has been well appreciated, PKC (namely, classical PKCs such as PKCα, βI/βII, γ)-dependent phosphorylation of EGFR on T654 promotes endocytic disappearance of the receptor from the cell surface and thus acts as an inhibitory signal to EGFR signaling [30,116]. In A431 and human embryonic kidney 293 (HEK293) cells, diacylglycerol kinase θ has been shown to counteract such PKC-dependent inactivation of EGFR, so that Y845 phosphorylation and other signaling events are maintained [117].

Insulin also promotes the transactivation of EGFR, involving the phosphorylation of Y845 and Y1173, but not Y1045, in rat perfused liver or primary hepatocytes [118]. These events, as well as subsequent ERK/MAPK activation, are sensitive to an NKCC1 cation-chloride co-transporter inhibitor, bumetanide, suggesting that insulin-induced swelling of the cells is involved. In fact, low osmolality alone induces EGFR and ERK/MAPK activation. In addition, integrin-dependent signaling (see also below), a known sensing system for osmolality, is implicated in Src activation and Y845 phosphorylation in this system [118].

### 3.2. Reactive Oxygen Species and Y845 Phosphorylation

In cultured renal tubular epithelial cells, chronic angiotensin II (AII) exposure-induced epithelial-to-mesenchymal transition (EMT) is reported to involve the phosphorylation of EGFR Y845 and caveolin-1, both of which depend upon reactive oxygen species (ROS)-induced Src activity [119]. This signaling pathway localizes to lipid rafts and serves as a prolonged signaling cue to activate ERKs/MAPKs, which are critical components for EMT, whereas the AII exposure also induces acute signaling that involves the conventional ligand-induced activations of EGFR and ERKs, which seem to be unrelated to the occurrence of AII-induced EMT. In rat vascular smooth muscle cells, SII-angiotensin, a triply mutated angiotensin octapeptide (Ser^1^, Ile^4^, Ile^8^), and AII have been shown to promote ERK/MAPK activation in a manner that depends upon the Src phosphorylation of Y845 of EGFR [120]. The stimulation of dopamine D_2_ receptor (D_2_R) by bromocriptine in PC12 cells ectopically expressing D_2_R (PC12-D_2_R) also induces transactivation of EGFR involving Src phosphorylation of Y845 and leads to further signaling to the PI3K/Akt pathway, cytoprotective responses against oxidative stresses and anti-apoptotic cell functions [121]. Bombesin and lysophosphatidic acid (LPA), both of which are activators for G-protein-coupled receptors (GPCRs), also stimulate, in a transactivation-dependent manner, the EGFR signaling involving Y845 phosphorylation in rat-1 cells expressing bombesin/gastrin release hormone receptor (BoR-15) [122]. Src-dependent phosphorylation of caveolin-1 and EGFR Y845 was shown to occur in ionizing radiation-treated human bronchial carcinoma cells, A549. These phosphorylation events seem to occur independently of each other; however, radiation-induced nuclear transport and activation of DNA repair machinery in the cells were shown to require both of these events [123,124].

UVB radiation was shown to promote the phosphorylation of multiple tyrosine residues of EGFR, including Y845, and subsequent activation of Akt and inactivation of Ras-ERK/MAPK in epidermal keratinocytes [125]. In rat liver epithelial cells, GN4, peroxisome proliferator-activated receptor ligand induced not only the Pyk2-p38 MAPK pathway but also the Y845 phosphorylation-induced Grb2-Sos-RAS-ERK pathway, the latter of which can be blocked by the application of either PP2 or the antioxidant *N*-acetyl-l-cysteine, suggesting that Src and ROS are involved in this signaling system [126]. In the cell proliferation and malignancy of MCF7 and SUM149 breast cancer cells, Src-dependent (*i.e.*, dasatinib-sensitive) Y845 phosphorylation (*i.e.*, Y845F mutation-sensitive) signaling was shown to require the activity of p38 MAPK, but not the activity of the EGFR/kinase ERK/MAPK, as well as Akt [127]. ROS that are produced by colchicine and nocodazole, both of which disrupt microtubules, have also been reported to promote Y845 phosphorylation in vascular smooth muscle cells, R22, by which ERK/MAPK signaling is triggered to activate plasminogen activator inhibitor-1 (PAI-1) expression [128]. Functional interaction between uPAR and wild-type EGFR has been shown in Chinese hamster ovary-K1 cells [129] and in murine embryonic fibroblasts [130], in which Y845F mutation of EGFR or Src inhibition impairs the uPA-induced signaling events, such as STAT5b activation and cell proliferation. The uPAR-Src-EGFR Y845 pathway also contributes to fibronectin matrix assembly and integrin activation in foreskin fibroblast A1-F [131]. The neuronal function of Y845 phosphorylation has been demonstrated by Goldshmit *et al*. [132], who showed that it plays an important role in neurite outgrowth that is induced by the suppressor of cytokine signaling 2/SOCS2 in central nervous system neurons and in cultured PC12 cells.

The cytotoxicity of arsenic trioxide in keratinocytes involves NADPH oxidase/p67^phox^-mediated production of ROS, which is under the control of Src activation and Y845 phosphorylation of EGFR [133]. ROS-induced Y845 phosphorylation has also been demonstrated in mammary epithelial cells that are treated with benzo[*a*]pyrene 3,6-quinone and benzo[a]pyrene 1,6-quinone [134]. Survival of hepatocytes in the presence of cadmium also involves a mechanism mediated by NADPH oxidase, the activation of which in turn activates Src, eventually resulting in EGFR Y845 phosphorylation [135].

### 3.3. Involvement of Y845 Phosphorylation in Cell Cycle Control and Cell Viability

In some cell systems, Y845 phosphorylation seems to promote growth arrest and/or inhibition of proliferation. In A431 epidermoid carcinoma cells, growth arrest induced by high concentrations of EGF is accompanied by Y845 phosphorylation and up-regulation of the STAT3-p21^waf1^ pathway. In this case, sequestration of the phosphorylated Y845 signal by the introduction into the cells of an antibody that binds to Y845 attenuated these events [49]. A similar anti-proliferative function was also demonstrated in MB-231 breast cancer cells, in which angiocidin and EGF promoted the Y845 phosphorylation and activation of NFκB, which resulted in the induction of p21^waf1^ and cell cycle arrest [136]. Activation of EGFR signaling sometimes involves action of the extracellular signal that stimulates the shedding of the EGFR ligand. Liu *et al*. [137] have reported that thrombospondins, and their recombinant proteins containing EGF-like repeats, are capable of inducing EGFR activation (involving the phosphorylation of Y845 and other tyrosine residues) and cell migration in A431 cells through the activation of matrix metalloprotease 9 (but not through direct binding to EGFR). In A431 cells, in which EGFR is overexpressed, depletion of cholesterol with methyl-β-cyclodextrin causes site-specific phosphorylation of EGFR, including Y845 [138]. Further characterization of this event has revealed that cholesterol most likely affects the phosphorylation state of EGFR by perturbing membrane properties to form membrane microdomains or “lipid rafts”, where Src and other signaling proteins are enriched and constitute a platform for a variety of signal transductions [139–143]. Lipid rafts in vascular smooth muscle cells [144] and bladder carcinoma cells [89,90] have also been reported to work as a scaffold for Src-EGFR signaling. In the case of bladder carcinoma cells, disruption of the membrane microdomains by methyl-β-cyclodextrin results in the interference with Src-dependent signal transduction and the promotion of apoptosis under serum-deprived culture conditions [89,90], although its relationship to the phosphorylation state of EGFR (*i.e.*, Y845) is unknown.

The anti-apoptotic mechanism of cell proliferation often involves Y845 phosphorylation. In microvessel endothelial T2 cells, serum-deprivation-induced apoptosis can be prevented by TGFβ1, which promotes such EGFR signaling [145]. Knockdown of EGFR results in the decrease in TGFβ1-induced expression of PAI-1, a major TGFβ1 target that is required for the anti-apoptotic mechanism. TGFβ1-induced expression of PAI-1 in vascular smooth muscle cells, R22, has also been shown to require the Src phosphorylation of Y845 of EGFR [146].

EGFR function, as judged by Y845 and Y1068 phosphorylations, has also been implicated in the anti-apoptosis of PC-3 prostate cancer cells, for which the combined application of β-phenylethyl isothiocyanate and curcumin leads to apoptotic cell death [147]. The activation of EGFR involving Y845 phosphorylation by neurotensin, which is secreted from LNCaP-derived neuroendocrine-like cells, was also shown in PC-3 cells [148]. This supports the idea that malignant prostate cancer cells develop in an environment in which neuroendocrine-differentiated cells secrete mitogenic ligands. Depletion of polyamine sometimes confers resistance to apoptosis, and Y845 phosphorylation is reported to be important in this process [149]. In this case, the kinase activity of EGFR is also required for anti-apoptosis, suggesting the possibility that EGFR and Src synergize in this process.

There are some reported examples of the pro-apoptotic function of EGFR Y845 phosphorylation. Reinehr *et al*. [150] described that, in hydrophobic bile salt-induced apoptosis of hepatocytes, Yes, a Src family PTK, associates with and phosphorylates EGFR on Y845, and contributes to activation of the CD95/Fas/Apo-1-dependent death pathway. In this system, the association of Yes with EGFR can be prevented by the inhibition of cAMP-dependent protein kinase/protein kinase A (PKA), suggesting that the cAMP-PKA pathway acts as anti-apoptotic machinery. Further characterization of this hepatocyte apoptosis system has revealed that death ligand (e.g., CD95 ligand)-induced apoptosis involves the production of ROS, a direct activator of Yes, by a signaling pathway that includes sphingomyelinase, ceramide, PKCζ, and p47^phox^[151]. In primary hepatocytes, prothrombin induces apoptotic cell death that is accompanied by the prothrombin-dependent degradation of integrin α5. In this system, it has been shown that prothrombin promotes the interaction between integrin α5 and EGFR, which in turn leads to tyrosine phosphorylation of both proteins including Y845 phosphorylation, and activation of c-Jun *N*-terminal kinase that is thought to be directly involved in the thrombin-induced apoptosis of hepatocytes [152].

### 3.4. Cell Adhesion Signaling and Y845 Phosphorylation

Integrin-mediated cell adhesion is reported to involve the assembly of a macromolecular complex containing integrin αβ dimers, EGFR, p130^cas^, and Src, in primary skin fibroblasts, ECV304 [153]. In this multimolecular complex, EGFR is phosphorylated in an EGF-independent manner on Y845, Y1068, Y1086, and Y1173, but not on Y1148, a major autophosphorylation site. The involvement of Src in these phosphorylations is not known; however, Src seems to be required for the recruitment of EGFR to the cell surface in response to integrin activation [153]. On the other hand, Wang *et al*. [154] have shown that, in squamous carcinoma cells, SCC12, the depletion of ganglioside GM3, which has been reported to be inhibitory to the integrin-dependent activation of EGFR, induces the phosphorylation of EGFR on Y845, Y1068, and Y1148, but not on Y1086 and Y1173. In this case, Src dependence is only evident in Y845 phosphorylation, whereas Y1068 and Y1148 phosphorylations seem to require EGFR and PI3K activities, respectively [154].

In NMuMG cells that are derived from epithelial cells of mouse mammary gland, fibronectin-induced cell adhesion promotes ligand-independent activation of EGFR involving Y845 phosphorylation and the recruitment of p120^RasGAP^ and p190^RhoGAP^, thereby leading to the inhibition of stress fiber formation as well as the stimulation of filopodium formation and priming to EMT [155,156]. In this system, the dominant-negative function of mutant EGFR, in which a dileucine motif (679-LL) in the juxtamembrane domain of EGFR is mutated to two alanine residues (679-AA) because of its failure to undergo fibronectin-induced Y845 phosphorylation, has been characterized.

In colon carcinoma cells (HT29 and SW480) and breast cancer cells (MCF-7), homophilic ligation of E-cadherin and its interaction with β-catenin are reported to be inhibitory to cell growth by reducing the frequency of cells entering S phase [157]. In this system, inhibition of EGFR signaling involving Y845 phosphorylation, but not EGFR autophosphorylation, and STAT5-dependent signal transduction, but not ERK/MAPK activation, are critical. Intracellular interaction between Cas and Src is also reported to be responsible for the activation of EGFR signaling involving Y845 phosphorylation and STAT5b translocation to the nucleus that contribute to the tamoxifen-resistant proliferation and survival of MCF7 cells [158]. Epithelial cell adhesion, migration, and wound healing in hydrogen peroxide-treated rabbit cornea also involve the Y845 phosphorylation of EGFR [159].

### 3.5. Sperm Functions and Y845 Phosphorylation

Some recent reports have shown that the Y845 phosphorylation of EGFR may be important for the biological functions associated with male gamete cells, sperm. The treatment of bovine sperm with EGF or ouabain, a specific inhibitor of Na^+^/K^+^-ATPase, has been shown to promote the acrosomal exocytosis of sperm cells, an event that needs to occur before the contact between sperm and egg at fertilization [160]. Ouabain is known to stimulate intracellular signal transduction through the binding to membrane-associated Na^+^/K^+^-ATPase, trans-activations of the EGFR (suggesting the involvement of Src), and the release of ROS from mitochondria. In fact, the ouabain-induced acrosomal exocytosis involves PKA/Src-dependent Y845 phosphorylation in EGFR [160]. Y845 phosphorylation has also been shown to occur in ram sperm in response to EGF-induced capacitation [161]. As opposed to the case mentioned above, PKA activity can also act as an inhibitory signal for the Y845 phosphorylation of EGFR. In primary rat hepatocytes cultured on collagen-containing gels, EGF-induced Src-dependent Y845 phosphorylation and cell proliferation are augmented by the addition of H-89, a specific PKA inhibitor [162].

### 3.6. Other Cellular Functions and Y845 Phosphorylation

In pancreatic β-cells, Y845 and Y1068 phosphorylations of EGFR are involved in EGFR activation in response to thyrotropin-releasing hormone, by which pancreatic β-cells undergo successful development and maturation [163]. In the renal cortical collecting duct cell line, M1-kidney CCD, the mineralocorticoid aldosterone acts as a critical hormone in the regulation of sodium, potassium, and proton fluxes. Under these conditions, Src-dependent Y845 phosphorylation acts as a signal of the crosstalk between aldosterone receptor and EGFR, which culminates in the activation of protein kinase D1, a kinase responsible for gene expression in response to aldosterone [164].

A study of the intracellular signaling response to environmental pollutants has revealed the involvement of Y845 phosphorylation. Zinc ions (Zn^2+^) have been shown to promote Y845 phosphorylation of EGFR, by which Zn^2+^-induced Ras activation is triggered in B82L fibroblast cells [165]. This effect of Zn^2+^ involves neither dimerization nor autophosphorylation of EGFR, and can be canceled by the Src-specific inhibitor PP2, but not by the EGFR inhibitor PD153035; however, PD153035 has been shown to interfere with ERK/MAPK activation [166]. Another kind of environmental pollutant, hexachlorobenzene, has also been shown to promote Src activation and Y845 phosphorylation of EGFR, which leads to the activation of STAT5b and the ERK/MAPK pathways and cell migration [167,168]. Iron-containing air pollution particles have been shown to activate NF-κB, a major trigger of an acute inflammatory response that would act on the pulmonary epithelial cell surface, via a pathway involving Src and EGFR [169].

Other cellular functions involving Y845 phosphorylation include wound-induced activation of the Src/EGFR pathway in corneal epithelial cells [170], intestinal cell proliferation and tumorigenesis that can be inactivated by the interaction of farnesoid X receptor with Src, leading to the inactivation of Src and the decrease in Y845 phosphorylation [171], and colon cancer proliferation that is stimulated by Src-mediated aryl hydrocarbon receptor and EGFR interactions, leading to Y845 phosphorylation and ERK/MAPK activation [172]. In primary normal human bronchial epithelial cells, sialyl Lewis X modification of EGFR involving Y845 phosphorylation contributes to wound-induced epithelial repair [173]. Another line of evidence shows that reduction of the expression of protein-tyrosine phosphatase-μ results in an increase in tyrosine phosphorylation of EGFR, including that on Y845, in response to airway epithelial injury [174]. Analysis of keratinocytes as a model system for analyzing cutaneous tissue repair also demonstrates the involvement of EGFR phosphorylation including Y845 [175]. In this case, EGFR is trans-activated by the kinin B(1) receptor system; however, the involvement of Src in this event is not known. Src-dependent Y845 phosphorylation also contributes to ERK/MAPK activation and proliferation of normal keratinocytes, which involves amphiregulin-mediated autocrine signaling of EGFR [176]. An alkylating agent, *N*-methyl-*N*′-nitro-*N*-nitrosoguanidine, has been shown to compete with EGF in the binding to EGFR, thus inhibiting the signaling pathway regulated by EGFR phosphorylation, including the basal level of Y845 phosphorylation [177]. The sensitization of EGF-induced signaling (e.g., intracellular Ca^2+^ release) in primary cultures of rat adrenal chromaffin cells and PC12 cells by bradykinin-dependent G-protein-coupled signal transduction involves Src activation and the Y845 phosphorylation of EGFR [178]. This study also demonstrated that cholesterol-enriched membrane microdomains serve as a platform for the Src-EGFR functional interaction, and that Y845F mutation results in a failure of signal transduction. In an attempt to elucidate the mechanism of nuclear translocation/localization of EGFR in cetuximab-resistant NSCLC cells (NCI-H226), Iida *et al*. [179] have shown that Y1101, but not Y845, in EGFR is a critical tyrosine residue that is phosphorylated by Yes and Lyn.

## 4. Molecular Insights into Y845 Phosphorylation and Its Applications

In an initial report on Y845 phosphorylation of EGFR by Src, it was noted that the phosphorylation requires not only the catalytic activity of Src but also the binding of EGF to EGFR [40]. This suggests that EGF-induced dimerization and/or other conformational changes of EGFR are required for Y845 phosphorylation. As mentioned above, Y845 is present in the activation segment of the protein kinase domain, a region that is located in-between the protein kinase subdomains VII and VIII. Protein-serine/threonine kinases as well as tyrosine kinases contain one or two phosphorylation sites in their own activation segment, most of which are autophosphorylation sites (e.g., PKA, Src) and/or kinase-activating phosphorylation sites (in some cases, they are phosphorylated by other kinases, e.g., EGFR, ERK/MAPK) [33,34]. Structural analyses of some protein kinases (e.g., cyclin-dependent protein kinase 1/CDK1, ERK/MAPK, PKA, Src) have demonstrated that phosphorylation in the activation segment leads to a conformational change in the catalytic cleft of the kinase domain, which allows the kinase domain to interact effectively with substrate proteins [180–186]. In the case of EGFR, the following scheme can be hypothesized: (1) binding of EGF to the extracellular domain of EGFR promotes a conformational change in its kinase domain (opening of the activation segment); (2) Y845 in the activation segment becomes exposed to the outer surface of the EGFR kinase domain (display of Y845); (3) exposed Y845 becomes accessible to Src, thereby being phosphorylated; and (4) the phosphorylated Y845 and its surrounding amino acid sequence provide a docking site for the Src homology 2 domain of Src and phosphotyrosine-binding domains of other signaling molecules (e.g., STAT3, CoxII), leading to the physical interaction of EGFR and these molecules.

Mutational studies on EGFR have shed light on the molecular insights into Y845 phosphorylation. Choi *et al*. [187] and Yang *et al*. [188] have shown that, in L834R and/or L837Q mutants of EGFR, which are pathologically found in NSCLC and 32D cells, Y845 phosphorylation takes place in an erlotinib-sensitive, but EGF- and Src-independent manner. Under these conditions, the activation of STAT5 and the expression of c-Myc are also stimulated, suggesting that the Y845 phosphorylation acts as in the case of its Src-dependent phosphorylation. Shan *et al*. [189] have studied further the structural impact of the L834R mutation of EGFR; it may counteract the disordered structure of the inactive EGFR kinase domain and facilitate the dimerization, so that the active kinase domain is stabilized. In addition, it has been suggested that Y845 phosphorylation, by acting as a more physiological trigger of the conformational change seen in the L834R mutant, is part of the self-sustained EGFR activation and amplification. A similar mechanism of stabilization of the active site by the amino acids near Y845 has also been reported by Timms *et al*. [190], who showed that E844 and H846 may participate in H-bonding interactions, thus stabilizing the active site region of the EGFR kinase domain. Another line of evidence using a synthetic peptide (EGFR-13), which corresponds to residues 645–657 of EGFR, has indicated that the EGFR-13 region interacts markedly with some other regions of the EGFR kinase domain, so that kinase activity of EGFR involving Y845 phosphorylation is stimulated [191]. These results suggest that EGFR has the potential to autophosphorylate on Y845 in an EGFR conformation-dependent manner. This idea is also supported by the study of Qiu *et al*. [192], who showed that a mutant EGFR, in which a stop codon had been introduced following the position encoding G998, could autophosphorylate Y845 in a PP2-insensitive and erlotinib-sensitive manner.

There are some reports showing that the phosphorylated Y845 sequence may be useful as a resource for artificial substances that could manipulate cellular functions. Buerger *et al*. [193] have reported the screening and identification of a short peptide aptamer that interacts with the kinase domain of EGFR and interferes with certain steps of EGFR signaling, including Y845 phosphorylation, Shc and STAT3 activations, and the growth of Herc cells (NIH3T3 overexpressing EGFR), SKBR breast cancer cells, and A431 cells, but not ERK/MAPK activation. The short aptamer peptide, termed KDI1, has an amino acid sequence of VFGVSWVVGFWCQMHRRLVC and its amino and carboxyl termini are coupled with bacterial thioredoxin protein that serves as a scaffold protein for the peptide. Kim and Huang [194] have reported that a synthetic peptide mimicking the EGFR Y845 site (EEEEpYFELV) can be successfully delivered into lung cancer cells, H460, and to the tumor in a xenograft mouse model, by which biochemical (e.g., STAT5 phosphorylation) as well as cell biological functions (e.g., tumor growth) of cancer are effectively blocked. We have also shown that an antibody, which recognizes a phosphoY845 region of EGFR, can be introduced into A431 cells, and that it acts as an inhibitor of EGF-dependent signal transduction, such as STAT3 activation and p21^waf1^ induction [49]. Thus, these materials designed for the Y845-containing region of EGFR are useful tools to investigate the cellular functions regulated by Y845, with a potential for further development as therapeutic reagents for cancer and other diseases.

## 5. Conclusions

**Figure 2:**
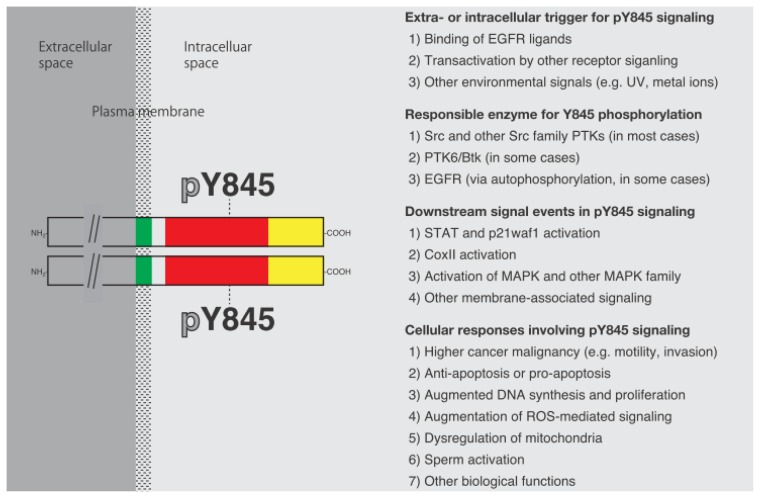
A sequence of events associated with Y845 phosphorylation of EGFR. Phosphorylation of EGFR on Y845 is usually catalyzed by Src and other Src family PTKs, and in some cases, is mediated by a non-Src family PTK or autophosphorylation of EGFR. The Y845 phosphorylation modulates a variety of cellular functions through the activation of several downstream events. It can also be used as a diagnostic marker of cancer treatments and for other applications that harness the phospho-Y845 signal into an antibody-, peptide-, or some other materials-based biological mimetics, as described in the main text.

For the past eighteen years or so, since the initial discovery of the Src phosphorylation of EGFR on Y845, much progress has been made in the recognition that Y845 phosphorylation takes place in a variety of cancerous and normal physiological cell contexts, as well as in the understanding that Y845 phosphorylation plays pivotal roles in these cellular contexts (Figure 2). Because EGFR and Src are expressed in almost all normal cells and tissues, the possible impact of their molecular interactions that lead to Y845 phosphorylation may not be limited to the known phenomena described in this review article, but rather spread more to other cellular functions that have not yet been documented. EGFR and Src are prototypical gene products, whose viral counterparts are oncogenic, and molecular and enzymatic functions are the transmembrane receptor PTK and the cytoplasmic non-receptor PTK, respectively. The co-overexpression of these proteins sometimes confers high malignancy for certain kinds of human cancer, and their continued study should provide more information of fundamental importance in the field of basic cell biology, as well as in cancer and other pathological areas of cell biology and medicine.
